# Spatial distribution of light-melanosome interaction dependent on irradiation fluence and spot size for short-pulsed laser skin treatment: A phantom study

**DOI:** 10.1007/s10103-025-04430-x

**Published:** 2025-05-30

**Authors:** Gakuto Takeda, Yu Shimojo, Toshiyuki Ozawa, Takahiro Nishimura

**Affiliations:** 1 Osaka University, Osaka, Japan; 2 Osaka Metropolitan University, Osaka, Japan; 3 Japan Society for the Promotion of Science, Tokyo, Japan

**Keywords:** Short-pulsed laser, Pigmented lesion, Melanosome, Phantom

## Abstract

Short-pulsed lasers provide safe and efficacious treatment of pigmented lesions by setting irradiation parameters based on immediate whitening resulting from vacuolization. However, visual observation of this phenomenon as an irradiation endpoint is subjective and makes it difficult to assess the depth to which the light response is induced inside the skin. To quantitatively understand the dependence of the light response on irradiation parameters, this study analyzes the spatial distribution of light-melanosome interaction based on the fluence distribution in an optical phantom. Nanosecond laser irradiation was applied to an optical phantom with a uniform distribution of melanosomes with varying irradiation fluence and spot size. The observed spatial distribution of the light-melanosome response was compared with the fluence distribution in the phantom calculated from numerical simulations. The resulting relationship between irradiation parameters and optical response showed that cavitation increased rapidly at an in-phantom fluence of 1.37 J/cm$^2$ and reached a saturated state at the threshold fluence for melanosome disruption. Additionally, increasing the spot size promoted cavitation at greater depths as well as increasing irradiation fluence, highlighting the need to consider both irradiation fluence and spot size for treatment depth control. The experimental results suggest the potential for quantitative control of optical responses in laser treatment of pigmented lesions. Further validation by experiments on samples with spatial distribution of cutaneous melanosomes is required.

## Introduction

Short-pulsed lasers offer a minimally-invasive and effective approach for treating pigmented skin lesions [1, 2]. Nanosecond lasers can minimize heat diffusion to surrounding tissues because their pulse widths are shorter than the thermal relaxation time of melanosomes [3]. Melanosomes are cellular organelles surrounded by a lipid bilayer that synthesize, store, and transport melanin pigments, and are mainly found within melanocytes. Selective laser treatment of lesions requires irradiation wavelengths preferentially absorbed by melanosomes and irradiation fluence sufficient to induce melanosome disruption [4, 5]. Notably, irradiation fluence must be appropriately adjusted on the basis of the lesion’s depth [6] because melanosome distribution varies depending on the type of lesion [7]. Excessive fluence increases the risk of complications such as scarring and post-inflammatory hypopigmentation [8]. Therefore, it is essential to devise a laser irradiation method capable of inducing spatially selective light responses to melanosomes according to lesion location.

When laser light penetrates the skin and reaches the lesions, melanosomes absorb the light, resulting in vacuolization and then generation of tiny cavities around the melanosomes [9]. These cavities increase light scattering in the skin, causing immediate whitening, which is frequently observed in the basal layer where melanocytes are abundant. These phenomena serve as a visual endpoint for laser irradiation [10]. However, this endpoint heavily relies on the surgeon’s experience and skill [10]. Additionally, in the dermis, where lesions may reside deeper, quantitatively assessing the depth of induced light responses remains challenging because laser light propagates in the tissue through scattering and absorption, attenuating the fluence with increasing depth. The degree of light-melanosome interaction is higher in areas with elevated fluence and lower in areas with diminished fluence. It is therefore necessary to analyze the degree of light response depending on fluence distribution in the tissue.

In this study, we evaluate the spatial distribution of light-melanosome interaction relative to irradiation fluence and spot size by analyzing the degree of cavitation induced by laser irradiation based on fluence distribution in the tissue. A 532 nm nanosecond laser was used in this experiment because it is widely used in the treatment of pigmented lesions [11]. Optical phantoms are used for this analysis. Phantom experiments can provide a reproducible experimental environment [12]. In addition, they allow easier control of conditions such as optical properties as opposed to using biological tissue samples. In this experiment, laser irradiation is applied to phantoms with uniformly distributed melanosomes, and their light responses are observed. The relationships between irradiation parameters and light-melanosome interaction are then quantitatively evaluated by comparing the spatial distribution of tiny cavities with the fluence distribution obtained through light propagation simulations. These results are expected to provide clinical reference for setting irradiation parameters to control the range of light-melanosome interaction in the skin.

## Materials and methods

### Phantom preparation

Optical phantoms were created using melanosomes extracted from porcine eyes as substitutes for human skin melanosomes. Porcine eyes were purchased from Tokyo Shibaura Zoki. To extract melanosomes, the surrounding adipose tissue and vitreous humor were removed from the porcine eyes, and the retinal layer was carefully separated [13]. The detached retinal layer was rubbed with a cotton swab in $70\,\textrm{ml}$ of distilled water to disperse the melanosomes into the solution. The melanosome-dispersed liquid was filtered using filter paper with a particle retention size of $2.7\,\mathrm {\mu m}$, and the filtrate was centrifuged at $3,000\,\textrm{rpm}$ for $20\,\textrm{minutes}$. The supernatant was slowly aspirated with a micropipette to remove cellular debris and blood, thereby isolating the melanosomes. Distilled water was then added to the extracted melanosomes to prepare a suspension.

The absorbance of the suspension was measured using a spectrophotometer (U-3000, Hitachi), yielding an absorbance of 1.71 at a wavelength of $532\,\textrm{nm}$. Intralipid (Intralipos Infusion 20%, Otsuka Pharmaceutical) was added to this suspension as a scatterer to achieve a final concentration of 2 vol%, followed by the addition of 10% gelatin (G2500-500G, SIGMA). The mixture was stirred at 40 ${}^\circ$C for 10 minutes. The prepared solution was poured into molds made with either Petri dishes or slide glasses (S112, MATSUNAMI) paired with 1-mm-thick spacers and then cooled at 4 ${}^\circ$C for $3\,\textrm{hours}$. Phantoms prepared with Petri dishes were used for laser irradiation experiments, while those prepared with slide glasses were used for optical property measurements. Five phantoms were prepared for each experiment.

The optical properties of the phantoms were measured using a double-integrating-sphere system and the inverse Monte Carlo (IMC) method. The absorption and reduced scattering coefficients were determined to be $0.348 \pm 0.006$ $\mathrm {mm^{-1}}$ and $2.81 \pm 0.24$ $\mathrm {mm^{-1}}$ at a wavelength of 532 nm, respectively. The anisotropy factor and refractive index used for the IMC analysis were 0.86 and 1.4, respectively [14].

### Laser irradiation experiment

The phantoms were irradiated with pulsed light using a clinical nanosecond laser with a wavelength of 532 nm and a pulse width of 50 ns (Alex TriVantage, Candela). The irradiation conditions were fluences of 2, 3, 4, and 5 J/cm$^2$, a spot size of 2 mm, an output stability of $\pm 20\%$, a circular top-hat beam profile, and single-shot operation. These parameters were based on nominal values provided by the manufacturer for human skin. The laser beam was directed perpendicularly to the phantom surface, with the handpiece fixed at a constant distance from the sample surface to the radiation aperture. To prevent backscattering, black aluminum foil (BKF12, Thorlabs) was placed beneath the phantom. Five locations on the prepared phantoms were irradiated. After irradiation, the phantoms were sectioned at the beam center using a scalpel, and the cross-sections were covered with glass coverslips for imaging. Imaging was performed using a complementary metal-oxide-semiconductor camera (BFS-U3-23S3, FLIR) and a camera lens (MLM-3XMP, Computar). All experiments were conducted at room temperature.

### Monte Carlo simulation of light propagation

Light propagation simulations were performed using Monte Carlo eXtreme to calculate fluence distributions in the phantoms [15]. The Monte Carlo method is a probabilistic approach that simulates photon trajectories using random sampling to model light transport in scattering media. By tracing the interactions of a large number of photons, this method provides an accurate estimation of fluence distributions within the phantom. The number of photon packets was set to $10^9$. The tissue model consisted of a cubic voxel grid of $550 \times 550 \times 550$ elements with a voxel size of $0.1\,\textrm{mm} \times 0.1\,\textrm{mm} \times 0.1\,\textrm{mm}$. The model assumed a single-layer phantom structure and incorporated the obtained absorption and reduced scattering coefficients, an anisotropy factor of 0.86, and a refractive index of 1.4. The *y*-axis was aligned with the depth direction, with $y=0\,\textrm{mm}$ corresponding to the phantom’s bottom surface. The model was centered on the *xz*-plane at $(x, z) = (2.75\,\textrm{mm}, 2.75\,\textrm{mm})$. The beam profile mimicked the laser irradiation experiment, with a 2-mm top-hat intensity distribution and irradiation fluences of 2, 3, 4, and $5\,\mathrm {J/cm^2}$. The fluence distribution in the cross-section containing the optical axis was output and overlaid with cross-sectional images of the phantoms to compare the fluence distribution with the spatial distribution of tiny cavities. Additionally, light propagation simulations were performed for spot sizes of 3 and $5\,\textrm{mm}$ at a fluence of $2\,\mathrm {J/cm^2}$ to compare these results with the numerical results for a 2-mm spot size. For the 5-mm spot size, a cubic voxel grid of $700 \times 700 \times 700$ elements was used with the model center set at $(x, z) = (3.50\,\textrm{mm}, 3.50\,\textrm{mm})$. The other conditions were consistent with the simulations for spot sizes of 2 and $3\,\textrm{mm}$.

### Comparison of cavitation regions and fluence distribution

**Fig. 1:**
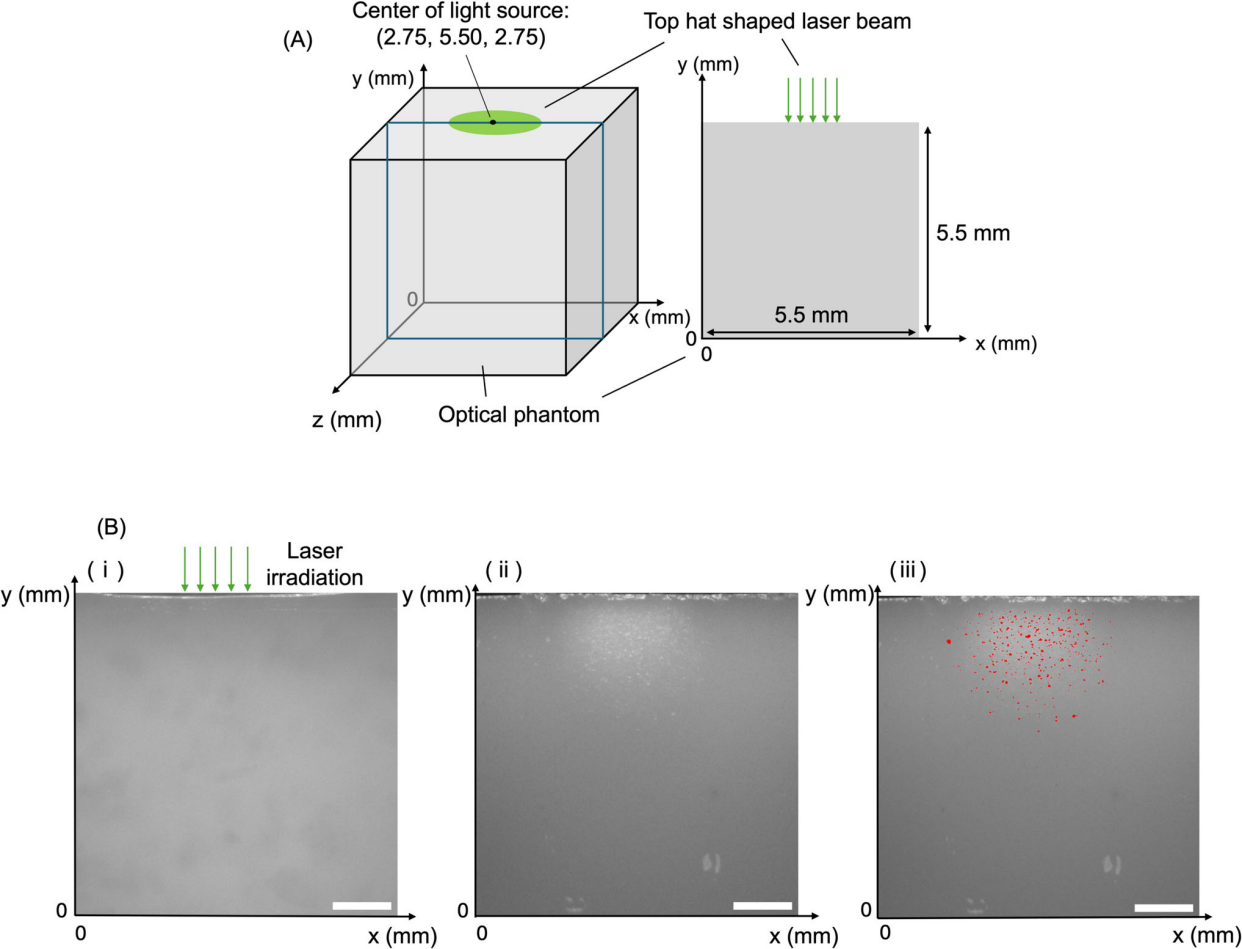
(A) Schematic of phantom geometry used in Monte Carlo simulation. (B) Cross-sectional images of the melanosome phantom. (i) Phantom before laser irradiation. (ii) Phantom after laser irradiation. (iii) Overlay of a cross-section of the irradiated phantom and the tiny cavities extracted from the image (indicated in red). Scale bars: $1\,\textrm{mm}$

To extract cavity regions from cross-sectional images of the phantoms after laser irradiation, grayscale conversion was performed using ImageJ [16]. In cavity regions, light scattering resulted in higher brightness than in the surrounding tissue. Regions where brightness exceeded the local average by 10 or more within a circular area of radius 0.1 mm were defined as cavity regions. To remove noise, the same processing was applied to pre-irradiation phantom cross-sectional images, and cavities smaller than the average size in the pre-irradiation phantom were removed from the post-irradiation cavity regions. A 0.19-mm depth range from the phantom surface was excluded from the analysis because of deformation during sectioning. This range was determined from the maximum depth of unmeasurable areas across 20 samples. A representative image of the resulting cavity regions is indicated in red in Fig. 1(C). The cross-sectional images of fluence maps and cavity regions were aligned by matching the centroids of the optical axis in the simulation and the spatial distribution of the tiny cavities. From this, the ratio of the cavity area to the fluence area was plotted with respect to fluence in increments of $0.1\,\mathrm {J/cm^2}$ to analyze the relationship between fluence and cavity formation. This enabled analysis of the variation in cavitation with respect to fluence. Furthermore, to quantitatively evaluate the threshold fluence for cavitation onset, the degree of cavity formation with respect to fluence was fitted using the following sigmoid function:

1 $$\begin{aligned} y = \frac{L}{1 + \exp (-k(x - x_0))} + b, \end{aligned}$$

where *L* is the maximum response (saturation cavitation ratio), $x_0$ is the threshold fluence for cavitation onset, *k* is the slope of the curve, and *b* is the baseline offset . Fitting was performed using the least-squares method.

## Results

### Analysis of phantom cross-sections after laser irradiation

Figure 1 shows the cross-sectional images of the phantom before and after laser irradiation, as well as following the cavity extraction processing (irradiation fluence: $2\,\mathrm {J/cm^2}$). In all three images, the upper edge corresponds to the phantom surface, with the laser beam radiating in the direction indicated by the arrow (Fig. 1A). No tiny cavities were observed in the phantom prior to laser irradiation (Fig. 1A). However, cross-sectional analysis post-irradiation revealed the formation of tiny cavities (Fig. 1B). Artifacts, such as brightness saturation caused by light reflection from irregularities formed on the upper surface during sectioning, were excluded from the analysis. Figure 1C shows the phantom after laser irradiation overlaid with the extracted cavity regions. The cavity density was highest near the optical axis and gradually decreased with increasing depth and distance from the axis.

### Comparison of cavitation regions and fluence distribution

**Fig. 2:**
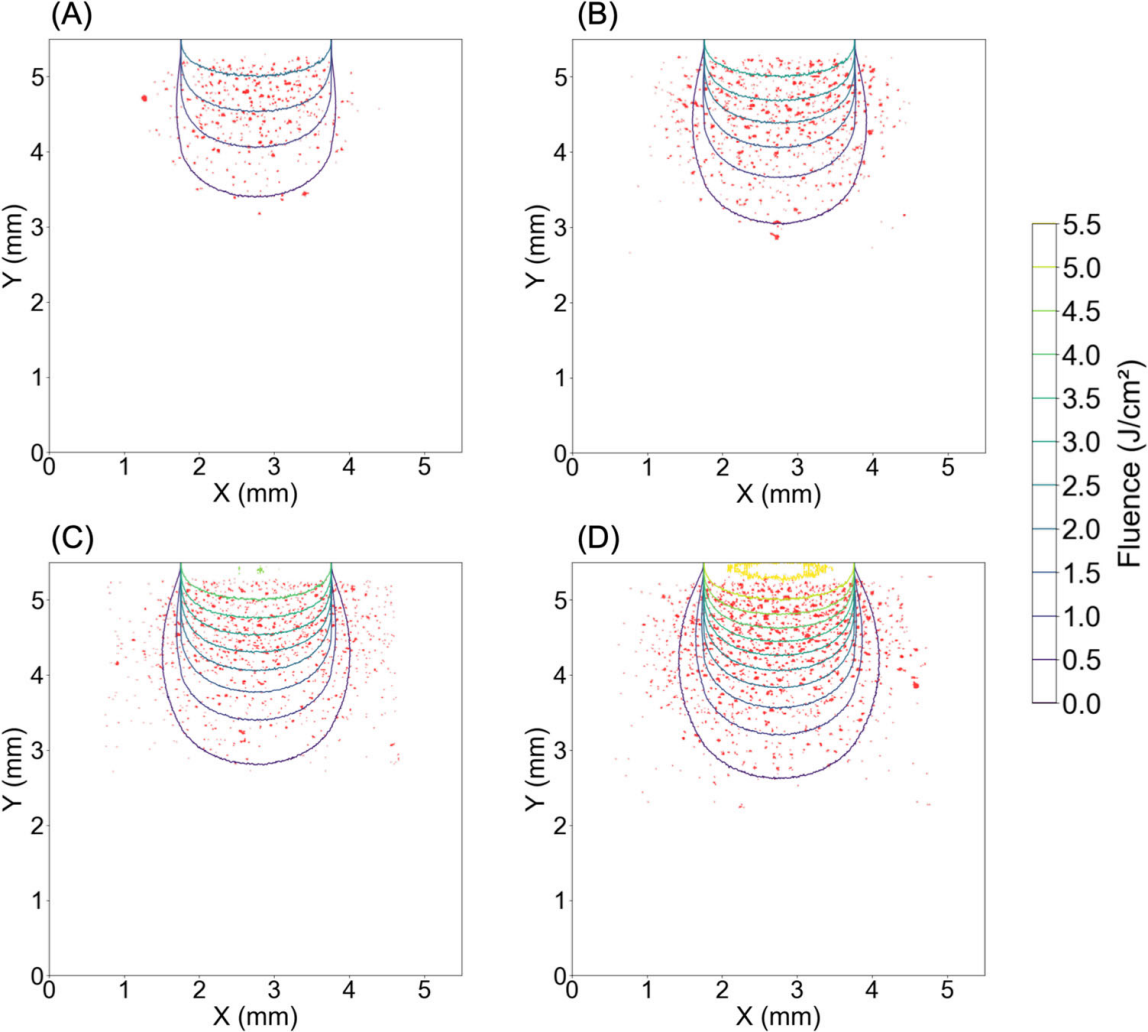
Overlay of fluence distributions in the phantom and cavity regions. Irradiation fluences are (A) $2\,\mathrm {J/cm^2}$, (B) $3\,\mathrm {J/cm^2}$, (C) $4\,\mathrm {J/cm^2}$, and (D) $5\,\mathrm {J/cm^2}$. The contour lines show the calculated fluence distributions with 0.5-J/cm$^2$ intervals

**Fig. 3:**
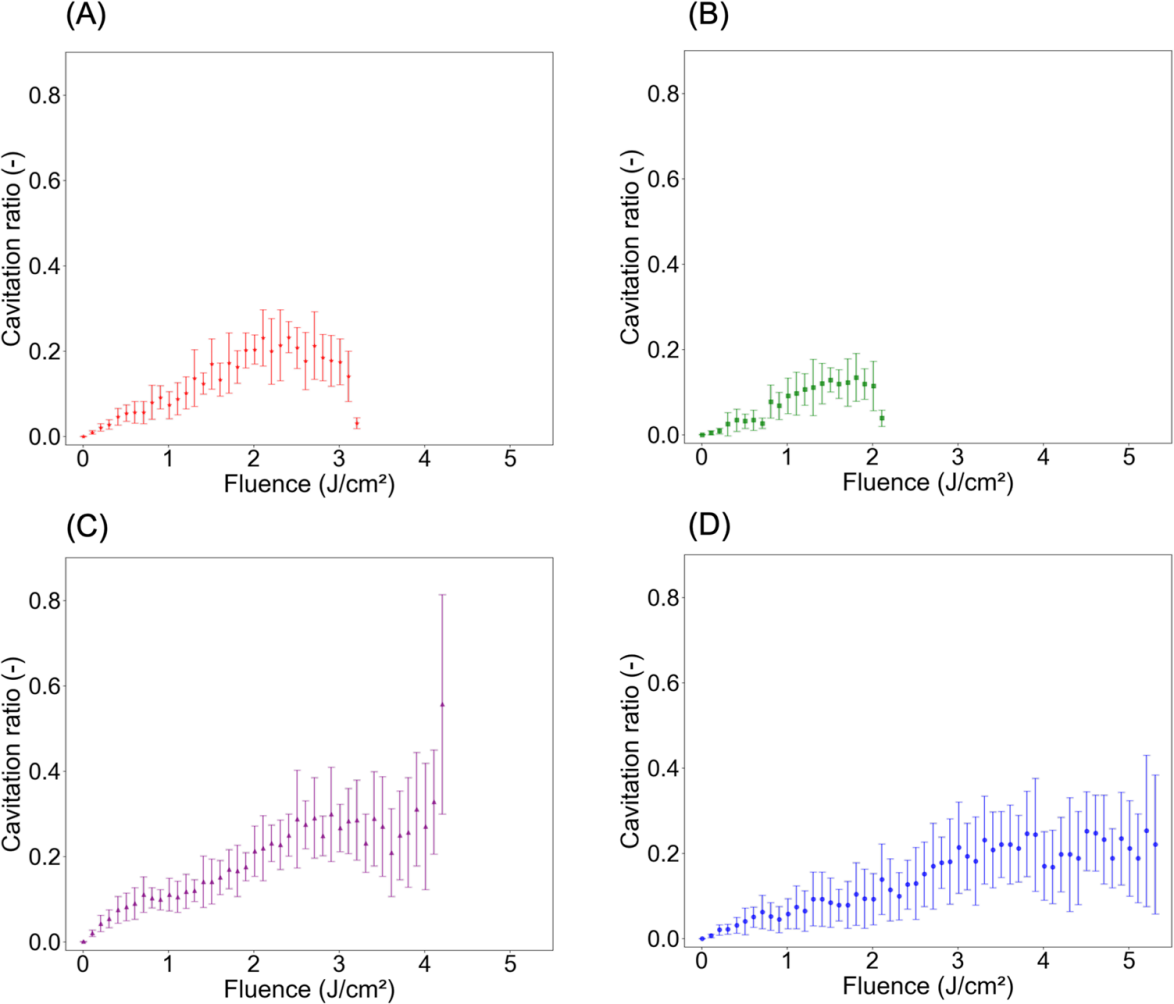
Relationship between fluence distribution in the phantom and cavity ratio calculated as the cavity area divided by the fluence area when the fluence width is set to $0.1\,\mathrm {J/cm^2}$. Irradiation fluences are (A) $2\,\mathrm {J/cm^2}$, (B) $3\,\mathrm {J/cm^2}$, (C) $4\,\mathrm {J/cm^2}$, and (D) $5\,\mathrm {J/cm^2}$

**Fig. 4:**
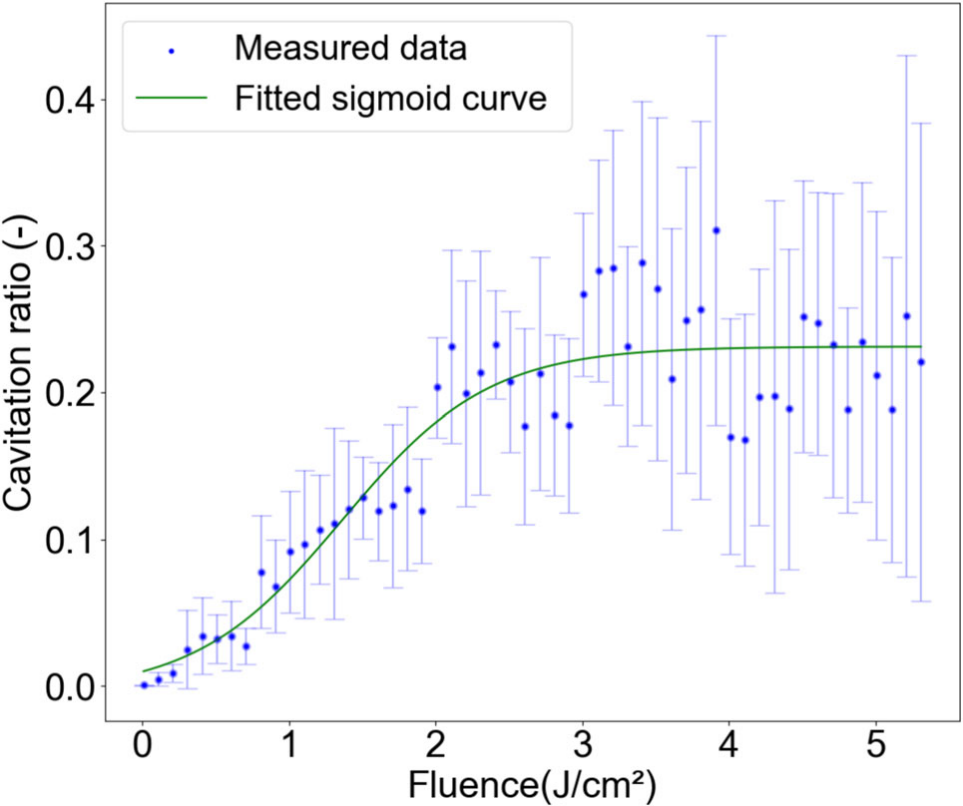
Sigmoid curve fitted to the experimentally measured data for the relationship between fluence distribution in the phantom and cavity ratio

Figure 2 shows the comparison between the fluence distribution within the phantom and the experimentally determined locations of cavitation. The calculated fluence distribution is represented using contour lines at 0.5-J/cm$^2$ intervals. The laser beam, affected by scattering, propagates diffusely. Near the surface, the contour lines are closely spaced, indicating a high attenuation rate. Higher irradiation fluence results in greater cavity density and deeper cavitation formation. Moreover, in regions with higher fluence along the optical axis, the number of tiny cavities increases, and cavitation exhibits greater lateral spread compared with the depth direction. Figure 3 shows the cavitation ratio as a function of fluence within the phantom. This is calculated as the ratio of the cavitation area to the area corresponding to each fluence range, defined with a 0.1-J/cm$^2$ increment. Each error bar represents one standard deviation. The cavitation ratio increases with increasing fluence within the phantom, reaching saturation at fluences of 3-$5\,\mathrm {J/cm^2}$ (Fig. 3, C, D). Figure 4 shows the cavitation ratio alongside the fitted values. To address potential data variability in regions of low fluence, fitting was applied to specific ranges: irradiation fluences of $2\,\mathrm {J/cm^2}$, $3\,\mathrm {J/cm^2}$, $4\,\mathrm {J/cm^2}$, and $5\,\mathrm {J/cm^2}$ for fluences in the phantom of 0-$2\,\mathrm {J/cm^2}$, 2-$3\,\mathrm {J/cm^2}$, 3-$4\,\mathrm {J/cm^2}$, and 4-$5\,\mathrm {J/cm^2}$, respectively. The experimental values align closely with the sigmoid function fit. The resulting parameters are $(L, x_0, k, b) = (0.24, 1.37, 2.0, -0.005)$ with a threshold fluence for rapid cavitation increase at $1.37\,\mathrm {J/cm^2}$.

### Cavitation depth versus spot size and irradiation fluence

**Fig. 5:**
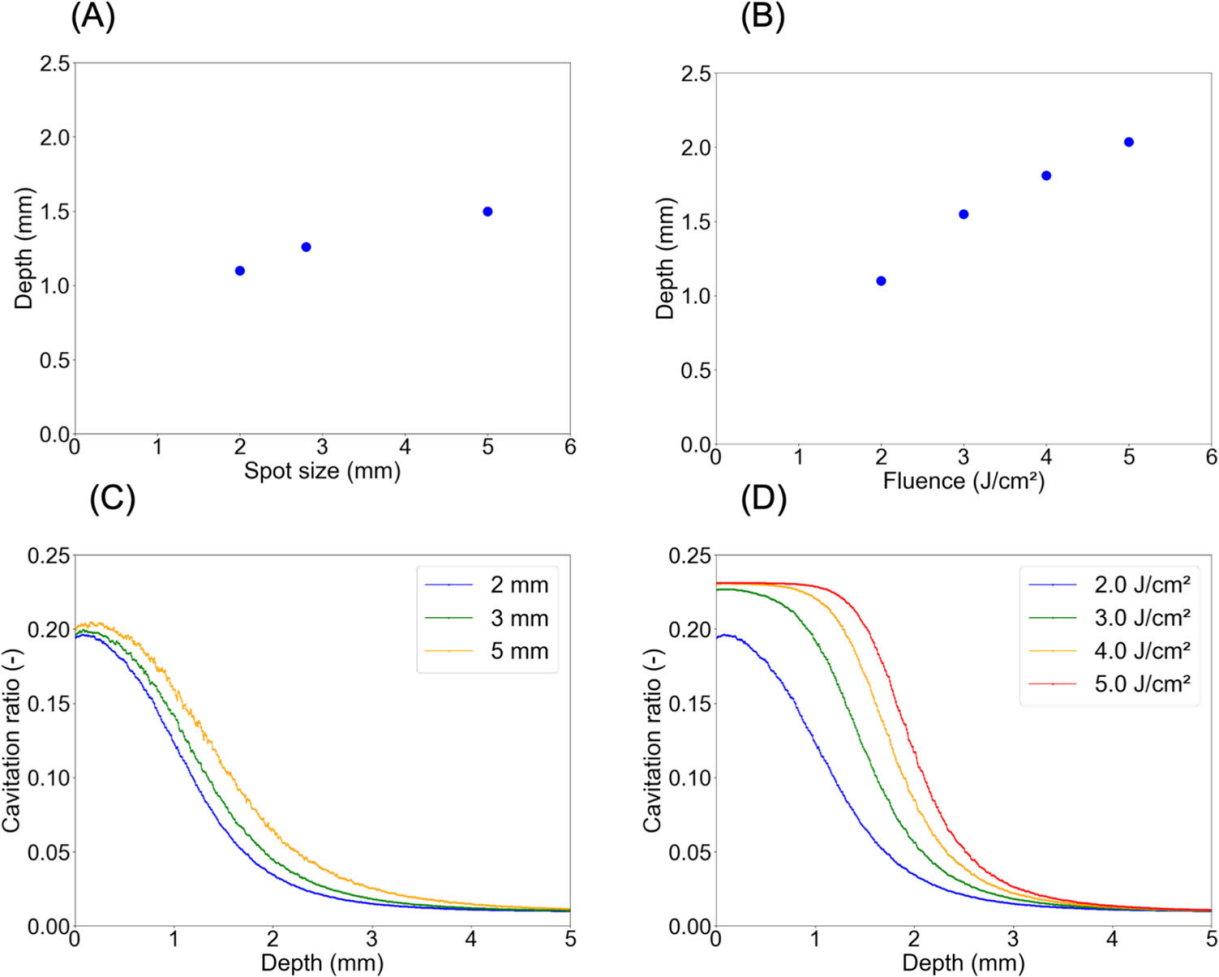
Simulation results depicting the depth at which the fluence threshold is reached along the optical axis. (A) Variation in spot size: 2, 3, and 5 mm, irradiation fluence of 2 J/cm$^{2}$ (B) Variation in irradiation fluence: 2, 3, 4, and 5 J/cm$^{2}$, spot size of 2 mm. Cavity ratio as a function of depth for (C) irradiation fluence of 2.0 J/cm$^{2}$ and spot sizes of 2, 3, and 5 mm and for (D) irradiation fluences of 2, 3, 4, and 5 J/cm$^{2}$ and spot size of 2 mm

Figure 5 (A, B) shows the simulated depth at which the fluence threshold for cavity formation ($1.37\,\mathrm {J/cm^2}$) is reached when the spot size is varied while maintaining a constant irradiation fluence. Increasing the spot size enabled irradiated laser light to penetrate deeper into the medium, even at the same irradiation fluence. Using the sigmoid function and the determined parameters, the cavitation ratio was calculated as a function of depth under two conditions: varying spot size with constant irradiation fluence and varying irradiation fluence with constant spot size (Fig. 5C, D). In Fig. 5(C), the cavitation at a particular depth increases with increasing spot size. This highlights that the probabilistic occurrence of light-melanosome interaction varies with depth even with constant irradiation fluence. In Fig. 5(D), higher irradiation fluence results in increased cavitation ratio at the same depth. These results confirm that the spatial distribution of light-melanosome interaction is significantly influenced by both the irradiation fluence and the spot size.

## Discussion

In this study, the spatial variations in light-melanosome interaction depending on spot size and irradiation fluence were quantitatively evaluated considering fluence distribution in the optical phantom. The experimental results confirmed that cavity density increased as irradiation fluence increased, with cavitation occurring even at greater depths. The simulation results demonstrated that spot size contributes to cavitation at deeper depths in addition to irradiation fluence. A comparison between the fluence distribution and the cavity regions revealed a sharp increase in cavitation at $1.37\,\mathrm {J/cm^2}$. This implies that the spatial distribution of light-melanosome interaction in the tissue may be controlled by appropriately managing irradiation fluence and spot size.

In this experiment, optical phantoms were used to analyze the spatial distribution of light-melanosome interaction based on the fluence distribution in the tissue. These phantoms exhibit low intra- and inter-sample variability, allowing for highly reproducible evaluation of the light-melanosome interaction. However, there are several differences between phantoms and biological tissues. Biological tissues exhibit distinct scattering and absorption coefficients that vary depending on individual differences and tissue type. Moreover, the phantoms used in this study had a uniform distribution of melanosomes, whereas human skin tissue tends to have a higher concentration of melanosomes in the epidermis and lesions. Real human skin also has multi-layered structures and blood vessels. These factors must be taken into account to translate the obtained relationship between light-melanosome interaction and fluence distribution into clinical applications. Therefore, future experiments should use biological tissues and a realistic model of human skin tissue to account for multi-layered structures and the spatial distribution of melanosomes and blood vessels. Combining these experiments with our results may help to provide insight into irradiation parameter settings to control the spatial distribution of light-melanosome interaction in the human skin.

This study determined the threshold fluence at which cavitation sharply increases, but this differed from the threshold at which cavitation actually occurs. The cavitation threshold for melanosomes under 532-nm, 10-ns laser irradiation has been reported as $0.0974 \pm 0.0007\,\mathrm {J/cm^2}$ [17]. The reported value was based on a probit analysis to determine whether cavitation occurred, which may cause the difference between the two thresholds. In an experiment with the same laser, the threshold fluence for melanosome disruption was reported as $3.0\,\mathrm {J/cm^2}$ [18]. At a fluence of $3.0\,\mathrm {J/cm^2}$, the cavitation ratio became constant at a high level, as shown in Fig. 4. Given that the thermal energy required for melanosome disruption is greater than that for cavitation [19], the validity of the threshold fluence for cavitation was confirmed. This implies that if irradiation fluence is set on the basis of tissue whitening due to cavitation, the fluence reaching the lesion may be insufficient to achieve melanosome destruction. Comparison of the cavitation region with the melanosome disruption region enables evaluation of the validity of the endpoint based on tissue whitening from the perspective of light-melanosome interaction.

To treat pigmented lesions, it is essential to adjust fluence according to the specific lesion characteristics. Ectopic Mongolian spots do not pose a recurrence risk, and therefore, structural disruption of melanosomes is required for effective treatment. Consequently, irradiation fluence exceeding the cavitation threshold must be delivered to the lesion. However, in lesions such as nevus of Ota, where melanocytes proliferate in the dermis, fluence sufficient to disrupt the melanocytes must be applied.

In this experiment, we focused on the mechanical response by melanosomes because the pulse width of the laser used is short enough to confine the heat within melanosomes and the thermal damage to the surrounding tissues is minimized. However, human skin has absorbers such as melanosomes in normal epidermis and blood vessels in normal dermis. The interaction of these absorbers with light has the potential to cause thermal damage to the surrounding tissues. This thermal effect is a critical consideration in clinical applications and can be addressed by histological examination of irradiated tissues [20] and thermal damage simulation [21].

To treat pigmented lesions, determining the spot size is often based on the lesion size [22], but increasing spot size results in widespread energy propagation, making cavitation more likely in deeper tissue layers. Thus, it is critical to adjust the spot size according to the depth of the target tissue. Irradiation fluence is also an important factor influencing cavitation. Cavities are more likely to form in deeper tissue layers at higher fluences, allowing for control over the light response in the depth direction. Therefore, appropriately setting the irradiation fluence can induce a light response specifically in the desired layer when targeting treatment at a particular depth. Thus, selecting the optimal combination of irradiation fluence and spot size may enhance treatment precision and spatially control cavitation depth according to tissue depth. Cavitation depth needs to be measured to be accurately controlled. Techniques such as optoacoustic tomography could be useful for this [23]. This method has the potential to visualize cavitation in tissue in real time non-invasively. Treatment safety and efficacy may be improved using this as feedback information for proper irradiation fluence and spot size settings.

## Conclusion

This phantom study analyzed the relationship between light-melanosome interaction and fluence distribution in tissue, quantitatively evaluating the impact of irradiation parameters on cavitation depth and ratio. The results showed that cavitation increased with increasing fluence, reaching a saturation point at the threshold fluence for melanosome disruption. Additionally, increasing spot size promoted cavitation in deeper layers, indicating that both irradiation fluence and spot size should be considered for effective depth control in treatment. This suggests the potential for quantitative control of the light response in laser treatment of pigmented lesions. Further studies using tissue samples will be necessary to validate the control of light responses when lasers are applied to skin tissues.

## Data Availability

No datasets were generated or analysed during the current study.
